# EphrinB2‐mediated chondrocyte autophagy induces post‐traumatic arthritis via rupture of cartilage homeostasis

**DOI:** 10.1111/jcmm.70095

**Published:** 2024-09-17

**Authors:** Zhengsheng Bao, Pinger Wang, Yanan Li, Huiqin Ding, Jingyuan Wen, Kaiao Zou, Xu Wang, Yang Yu, Xuefeng Li, Yingquan Liu, Hongting Jin, Lianguo Wu, Jun Ying

**Affiliations:** ^1^ Institute of Orthopedics and Traumatology The First Affiliated Hospital of Zhejiang Chinese Medical University (Zhejiang Provincial Hospital of Chinese Medicine) Hangzhou China; ^2^ The Second Clinical College Zhejiang Chinese Medical University Hangzhou China; ^3^ Department of Orthopedic Surgery The Second Affiliated Hospital of Zhejiang Chinese Medical University Hangzhou China; ^4^ Department of Orthopedic Surgery The First Affiliated Hospital of Zhejiang Chinese Medical University (Zhejiang Provincial Hospital of Chinese Medicine) Hangzhou China; ^5^ The First College of Clinical Medicine Zhejiang Chinese Medical University Hangzhou China

**Keywords:** EphrinB2, homeostasis, osteochondral, post‐traumatic arthritis

## Abstract

EphrinB2, a member of the Ephrin family, has been linked to several orthopaedic conditions. Nevertheless, the correlation between ephrinB2 and post‐traumatic arthritis (PTOA) remains unclear. Human PTOA cartilage from human and mouse knee joints was systematically analysed to investigate the relationship between EphrinB2 and PTOA using SO‐FG and toluidine blue staining, micro‐CT, histomorphometry, immunohistochemistry, immunofluorescence, lentiviral articular injection and in situ end labeling (TUNEL) assays. EphrinB2 expression was significantly downregulated in PTOA chondrocytes. Blocking EphrinB2 increased the breakdown of cartilage matrix in mice with PTOA via reducing the process of chondrocyte autophagy. The presence of severe cartilage damage was evident, as indicated by a considerable decrease in both cartilage thickness and area, accompanied by an increase in chondrocyte death. Altogether, EphrinB2 is required for the maintenance of cartilage homeostasis in post‐traumatic arthritis, and EphrinB2 ablation is associated with accelerated chondrocyte matrix degeneration, finally causing damage to the articular cartilage.

## 1 INTRODUCTION

Post‐traumatic arthritis (PTOA) refers to the development of osteoarthritis after a severe joint injury. This condition is characterized by significant morbidity and disability.^1^ PTOA is distinguished by discomfort and restricted functionality caused by the rapid deterioration of the cartilage in the joints.^2^ PTOA is known to develop in up to 87% of cases following an anterior cruciate ligament (ACL) rupture. This injury is considered the most major risk factor for PTOA, which is becoming more common. Approximately 252,000 ACL injuries are recorded annually in the United States, and there appears to be an increasing trend in these occurrences.^3^, ^4^, ^5^ It commonly occurs during sudden deceleration and direction change in non‐contact situations. Adolescents and young adults who participate in sports requiring pivoting and frequent direction changes have a high incidence of ACL injury. The risk in young women performing pivoting sports is 3–5 times higher than in men. The issue of PTOA has not received the necessary recognition. It deserves as a significant societal burden, with profound implications for the survival and employment prospects of young individuals. This could be attributed to a misunderstanding and lack of clarity, as well as the perception that it is an unavoidable aspect of the aging process.^6^

However, unlike osteoarthritis (OA), PTOA is induced by a specific injury that leads to extensive harm to the soft tissues within the joints, consequently accelerating the deterioration of the articular cartilage. This process leads to the programmed cell death and enlargement of chondrocytes, which then release various cytokines such as interleukin‐1 (IL‐1), tumour necrosis factor (TNF), nitric oxide synthase (NOS) and matrix metalloproteinases (MMPs). These factors ultimately lead to a reduction in the number of chondrocytes, deterioration of the extracellular matrix, and breakdown of the cartilage.^7^, ^8^ Therefore, it is essential to preserve chondrocyte homeostasis during PTOA. However, the molecular mechanisms responsible for the accelerated degradation of cartilage matrix metabolism in PTOA are still not fully understood.

Ephrins function as ligands for the Tyrosine kinase receptor, playing a significant role in the development of several organs and tissues, including the neurological and vascular systems.^9^, ^10^, ^11^ EphrinB2 is one of three essential EphrinB ligands and acts as a signalling protein that is attached to the cell membrane.^12^ Therefore, the process of osteoblast differentiation may be controlled and the formation of bone minerals can be enhanced by increasing the expression of EphrinB2.^13^ The overexpression of EphrinB2 in dental pulp MSCs resulted in an increased ability of osteogenic differentiation, leading to a more efficient healing process for alveolar bone defects.^14^, ^15^ Additionally, a study has found that specifically reducing the expression of EphrinB2 in chondrocytes resulted in abnormal bone and cartilage characteristics, including cartilage degradation, disruption of the cartilage hypertrophic zone, and irregular mineralization of the subchondral bone.^16^ However, the exact mechanism of cartilage degeneration remains unclear.

Autophagy is an intrinsic mechanism that is crucial for the replacement of organelles, therefore ensuring the stability of cells and controlling the destiny of cells.^17^ Dysregulated autophagy has been linked to the onset of various diseases. In the initial phases of OA, chondrocytes demonstrate heightened levels of autophagy, which subsequently decrease as the cartilage undergoes gradual degeneration.^18^, ^19^, ^20^ It has recently been discovered that the deletion of Ephrin B2 in osteoblasts causes the deregulation of many autophagy‐related genes, resulting in an increase in bone fragility.^21^ However, the effect of Ephrin B2 on autophagy in PTOA chondrocytes has not been studied.

EphrinB2 plays a role in multiple cellular and tissue processes, but its primary function is to control the modification of the extracellular matrix. Thus, the objective of this study is to elucidate the manifestation of EphrinB2 in chondrocytes in PTOA, evaluate its influence on the degradation of the cartilage matrix in PTOA, as well as its effect on the level of autophagy in chondrocytes, and investigate the underlying molecular mechanisms.

## 2 MATERIALS AND METHODS

### 2.1 Human cartilage collection

In this study, articular cartilage samples were collected from PTOA patients who underwent total knee arthroplasty at the Department of Orthopaedics, First Affiliated Hospital of Zhejiang University of Traditional Chinese Medicine. The study protocol was approved by the Ethics Committee of the First Affiliated Hospital of Zhejiang University of Traditional Chinese Medicine granted approval for all procedures(2019‐KL‐041‐01). All patients provided written informed consent to participate in the study. The human samples were steeped in a solution containing 4% paraformaldehyde (PFA), decalcified with 14% ethylenediaminetetraacetic acid to remove calcium deposits, embedded in paraffin and then performed to histological staining. The degree of cartilage deterioration was assessed using gross and histological findings, following the OARSI Osteoarthritis Cartilage histological Evaluation System.

### 2.2 Mice

10‐week‐old male C57BL/6J wild‐type mice were obtained from the Experimental Animal Research Centre of Zhejiang University of Traditional Chinese Medicine. In the case of anterior cruciate ligament transection (ACLT), the ACL was incised using microscissors following the exposure of the joint capsule. A lentivirus, containing 2 × 10^8^ transduction units, was injected into the knee joint 2 weeks after ACLT to suppress the expression of Ephrinb2 in chondrocytes in vivo. The mice were euthanized 4 weeks following either sham or ACLT surgery. The Animal Ethics Committee of Zhejiang University of Traditional Chinese Medicine granted approval for all animal handling methods conducted in this study (20221010‐20).

### 2.3 Micro‐CT, MRI and X‐ray analysis

A micro‐computed tomography (micro‐CT) scanner (SkyScan1176) was used to visualize and 3D image all specimens, with scanning parameters set at 45 kV, 500 μA and an exposure time of 770 ms. 3D reconstruction of the medial subchondral bone in tibial plateau and the entire knee joint was performed using software (SkyScan, CTVolx, v3.0). In addition, bone volume fraction (BV/TV) was measured using analysis software (SkyScan, CTAn, v1.15).

A GE 3.0 T Signa Excite superconducting MRI scanner with a 32‐channel body coil, a gradient field of 24 mT/m and a creep rate of mT/(ms) and a Siemens Verio 3.0 T superconducting MRI scanner with a 16‐channel phased array coil were used. An ultrashort echo time (UTE) pulse sequence (echo time 0.07 ms) was performed in the sagittal plane of the affected knee joint. Scanning parameters: slice thickness 0.8 mm, field of view 320 mm × 320 mm, matrix 160 mm × 160 mm, intra‐slice resolution 0.6 mm × 0.6 mm and excitation 2 times. Scanning software: NooPhase Wrap, Variable Bandwidth, Tailored RF.

For the diagnosis, a digital X‐ray machine was used, the manufacturer was Siemens (Brlliance CT), and the patient was instructed to adopt the supine position for the examination, and the joint and the tibia were photographed in the orthostatic position and in the lateral position, when photographing the lateral film, the current was set to 71 mA and the voltage to 58.5 kV, and when photographing the orthostatic film, the current was set to 71 mA and the voltage to 60 kV.

### 2.4 Histological observations

The knee specimens were submerged in a 4% solution of paraformaldehyde (PFA) for a duration of 3 days, followed by a decalcification process using disodium ethylenediaminetetraacetic acid (EDTA) at room temperature for a period of 2 weeks. Following paraffin embedding, tissue dehydration was carried out using a dehydrator. The paraffin samples were sliced in the sagittal plane with a thickness of 3.5 μm. Following the removal of wax and rehydration, the sections were subjected to staining with SO‐FG and toluidine blue in order to evaluate their morphology. Tartrate‐resistant acid phosphatase (TRAP) staining (Sigma–Aldrich, Missouri, USA) was performed according to the manufacturer's instructions. The OARSI score was utilized to evaluate the extent of articular cartilage deterioration in PTOA.

### 2.5 Immunohistochemistry and immunofluorescence stainings

The sections underwent deparaffinization, rehydration, immersion in a sodium citrate solution and were then heated in an oven at 60°C for 4 h to facilitate antigen retrieval. The sections were incubated with the primary antibody overnight at a temperature of 4°C. The next day, the sections were treated with the secondary antibody for a duration of 20 min or fluorescent secondary antibodies for 1 h at room temperature. A positive signal reagent (ZSGB‐BIO) was used to visualize the results, and the nuclei were stained with haematoxylin or DAPI. The antibodies utilized in this study were EphrinB2 (Huabio, ET1705‐33, China, 1:200, IHC/IF), Mmp13 (Abcam, ab39012, UK, 1:200, IHC), Col2 (Abcam, ab34712, USA, 1:200, IHC), Lc3B (Novusbio, NB‐100‐2220, USA, 1:100, IHC), p62 (Huabio, R1309‐8, China, IHC). The obtained images were subsequently quantified using Image‐Pro Plus 6. The program is developed by Media Cybernetics, located in Rockville, MD, USA.

### 2.6 In situ terminal labelling method

To detect apoptotic cells in articular cartilage, we conducted a TUNEL assay following the guidelines provided by the manufacturer (Beyotime, catalogue C1088). After removing the wax and rehydrating, the sections were briefly treated with deoxyribonuclease K‐free (20 μg/mL) at 37°C for 15–20 min to make them permeable. The sections were thereafter subjected to TUNEL reagent, and incubated at a temperature of 37°C in the absence of light for a duration of 1 h. Following this, the sections were restained with DAPI for a period of 10 min. Fluorescence microscopy was utilized to observe the position and distribution of DNA‐breaking indicators.

### 2.7 Cell culture

Primary articular chondrocytes were isolated from the femoral head cartilage of 2‐week‐old mice. The cartilage was broken down in a DMEM/F‐12 medium (Thermo Fisher Science). This mixture contained 0.3 mg/mL of collagenase P (Roche) and 1% penicillin/streptomycin (Thermo Fisher Science). The digested process continued 4 h at a temperature of 37°C, with occasional shock. The collected chondrocytes were seeded into 6‐ and 12‐well plates at densities of 10 × 10^5^ and 5 × 10^5^ cells per well, respectively. The cells were incubated in DMEM/F‐12 medium supplemented with 10% fetal bovine serum. Primary chondrocytes were exposed to recombinant mouse IL‐1β (SIGMA) at a concentration of 10 ng/mL for in vitro tests.

### 2.8 Real‐time PCR and western blot

Total RNA was extracted from primary chondrocyte cultures using TRIzol reagent (Sigma). The synthesis of cDNA was performed using the TaqMan Reverse Transcription Kit (Biomake). The reverse transcription‐polymerase chain reaction was conducted in accordance with the instructions provided by the manufacturer. The primer sequences for Mmp13, Col2, Actin, LC3B, P62, Beclin1 and GAPDH may be found in Table 1. Western blot analysis was conducted on protein lysates from primary articular chondrocytes employing antibodies. The primary antibodies utilized in this study were EphrinB2 (Huabio, ET1705‐33, China, 1:2000), Mmp13 (Abcam, ab39012, UK, 1:1000), Col2 (Abcam, ab34712, UK, 1:2000), Lc3b (Abcam, ab34712, UK, 1:2000), Lc3b (Novusbio, NB‐100‐2220, USA, 1:1000), P62 (Huabio, R1309‐8, China, 1:1000), Beclin1 (Abclonal, A7353, China, 1:1000), Caspase3 (Abcam, ET1608‐64, UK) and Bcl2 (Huabio, ET1702‐53, China, 1:2000).

**TABLE 1:** Primer sequences for qPCR.

Primer name	Sequence
Col2a1 forward	GGGAATGTCCTCTGCGATGAC
Col2a1 reverse	GAAGGGGATCTCGGGGTTG
Mmp13 forward	CTTCTTCTTGTTGAGCTGGACTC
Mmp13 reverse	CTGTGGAGGTCACTGTAGACT
BCL2 forward	5′‐CCTGTGGATGACTGAGTACCTG‐3′
BCL2 reverse	5′‐AGCCAGGAGAAATCAAACAGAGG‐3′
P62 forward	5′‐TGTGGAACATGGAGGGAAGAG‐3′
P62 reverse	5′‐TGTGCCTGTGCTGGAACTTTC‐3′
Beclin1 forward	5′‐GTGCGCTACGCCCAGATC‐3′
Beclin1 reverse	5′‐GTAGTGGAAGGTGGCATTGAA‐3′
β‐Actin forward	5′‐GGAGATTACTGCCCTGGCTCCTA‐3′
β‐Actin reverse	5′‐GACTCATCGTACTCCTGCTTGCTG‐3′

### 2.9 Statistical analysis

The study's results were presented as the mean value plus or minus the standard deviation. Statistical analysis was conducted using GraphPad Prism software, specifically version 8.0. The statistical analysis involved the use of two‐tailed unpaired parametric *t*‐tests to compare two groups, and multifactorial one‐way anova to compare multiple groups. Post‐hoc tests were conducted after the ANOVA. In order to conduct in vitro cellular investigations, observations were repeated independently a minimum of 3 times, and a significance level of *p* < 0.05 was used to determine statistical significance.

## 3 RESULTS

### 3.1 Downregulation of EphrinB2 expression in human PTOA cartilage

**FIGURE 1:**
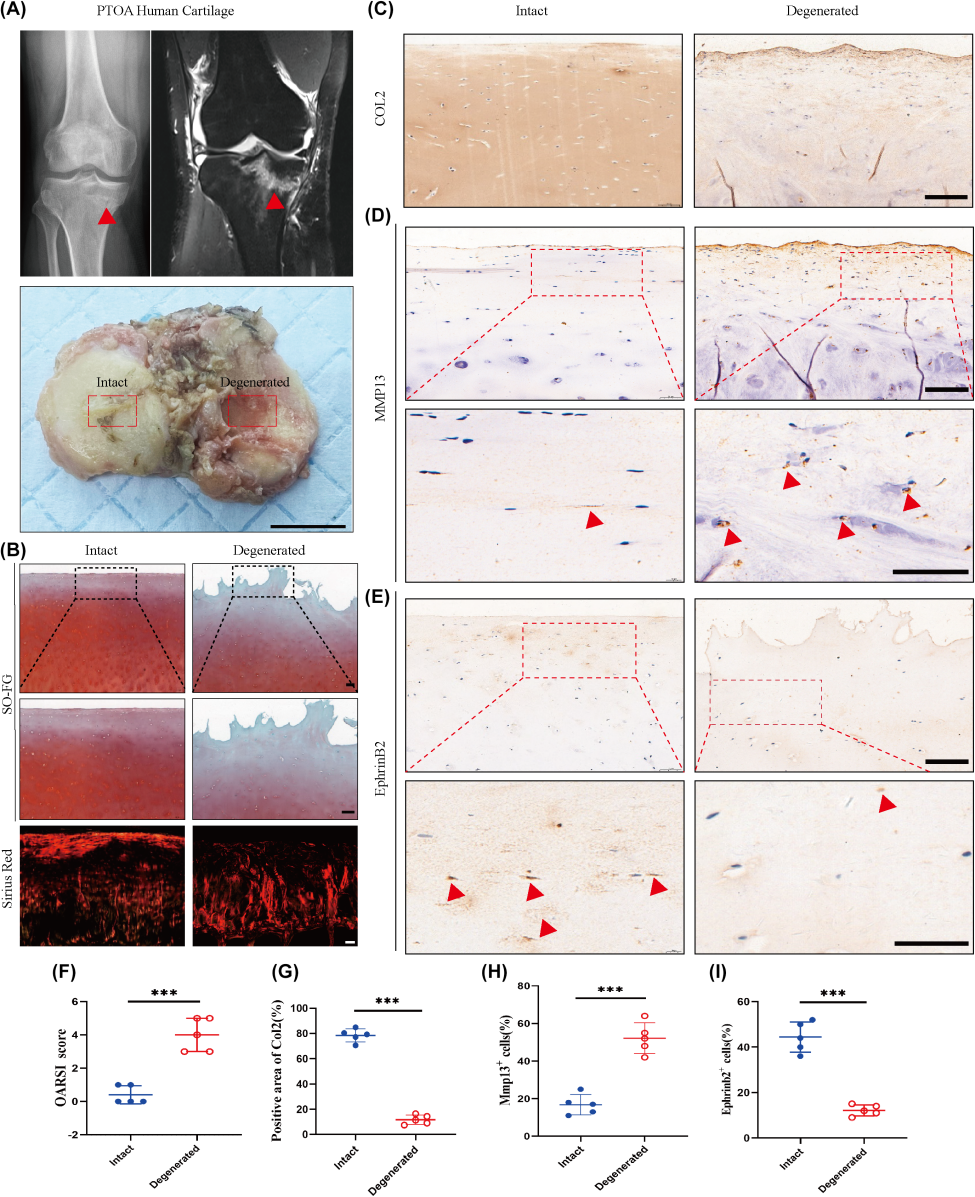
Expression of EphrinB2 in human post‐traumatic arthritis. (A) Human tibial plateau cartilage samples obtained from the patients who had undergone TKA surgery due to PTOA (*n* = 5 individuals) scale bar: 2 cm. (B) Representative images of intact and degenerated cartilage stained with Safranin O‐Fast Green and Pico‐Sirius Red. Scale bar:100 μm. (C) Representative immunohistochemistry staining for Col2 of intact and degenerated cartilage. Scale bar:100 μm. (D) Representative Mmp13 immunohistochemistry staining. Red arrows indicate positive cells. Scale bar:100 μm. (E) Representative immunohistochemistry staining for EphrinB2. Red arrows represent positive cells. Scale bar:100 μm. (F) OARSI score for assessing the degree of cartilage degeneration. (G) Quantification of Col2 positive cells in human cartilage. (H) Quantification of Mmp13 positive cells in human cartilage. (I) Quantification of EphrinB2 positive cells in human cartilage. All data were expressed as mean SD, (*n* = 5), *p < 0.05, **p < 0.01, ***p < 0.001.

To investigate the relationship between EphrinB2 and PTOA, we initially examined the expression of EphrinB2 in both intact and degenerated human cartilage samples. In this study, tibial plateau cartilage samples were taken from patients with post‐traumatic osteoarthritis who had undergone total knee arthroplasty (TKA). A visual examination revealed that the deteriorated cartilage after the injury primarily came from the medial tibial plateau, which had an eroded surface. On the other hand, the cartilage in the lateral region exhibited a smooth surface without roughness. We noticed a decrease in proteoglycan staining (red) in the deteriorated articular cartilage, which had a rough, nearly calcified surface (Figure 1A). Pico‐Sirius Red staining revealed that fibres arrangement was disorganized in PTOA cartilage, which was in contrast to the intact cartilage with aligned fibres in the Intact group (Figure 1B). Immunohistochemistry data showed that the expression of Col2 was decreased, as well as the increased expression of matrix metalloproteinase 13 (MMP13) in PTOA cartilage compared to healthy cartilage (Figure 1C,D,G,H). Furthermore, OARSI score was significantly increased in the PTOA group, as compared to the intact cartilage group (Figure 1F). The expression of Ephrinb2 in chondrocytes affected by trauma was also investigated, and it was found that chondrocytes in PTOA cartilage exhibit decreased expression of Ephrinb2 (Figure 1E,H). These data suggest a possible link between Ephrinb2 and the rapid breakdown of cartilage, the large decrease in chondrocyte numbers, and the accelerated degradation of the extracellular matrix that occurs in post‐traumatic arthritis.

### 3.2 EphrinB2 expression reduced in PTOA cartilage

**FIGURE 2:**
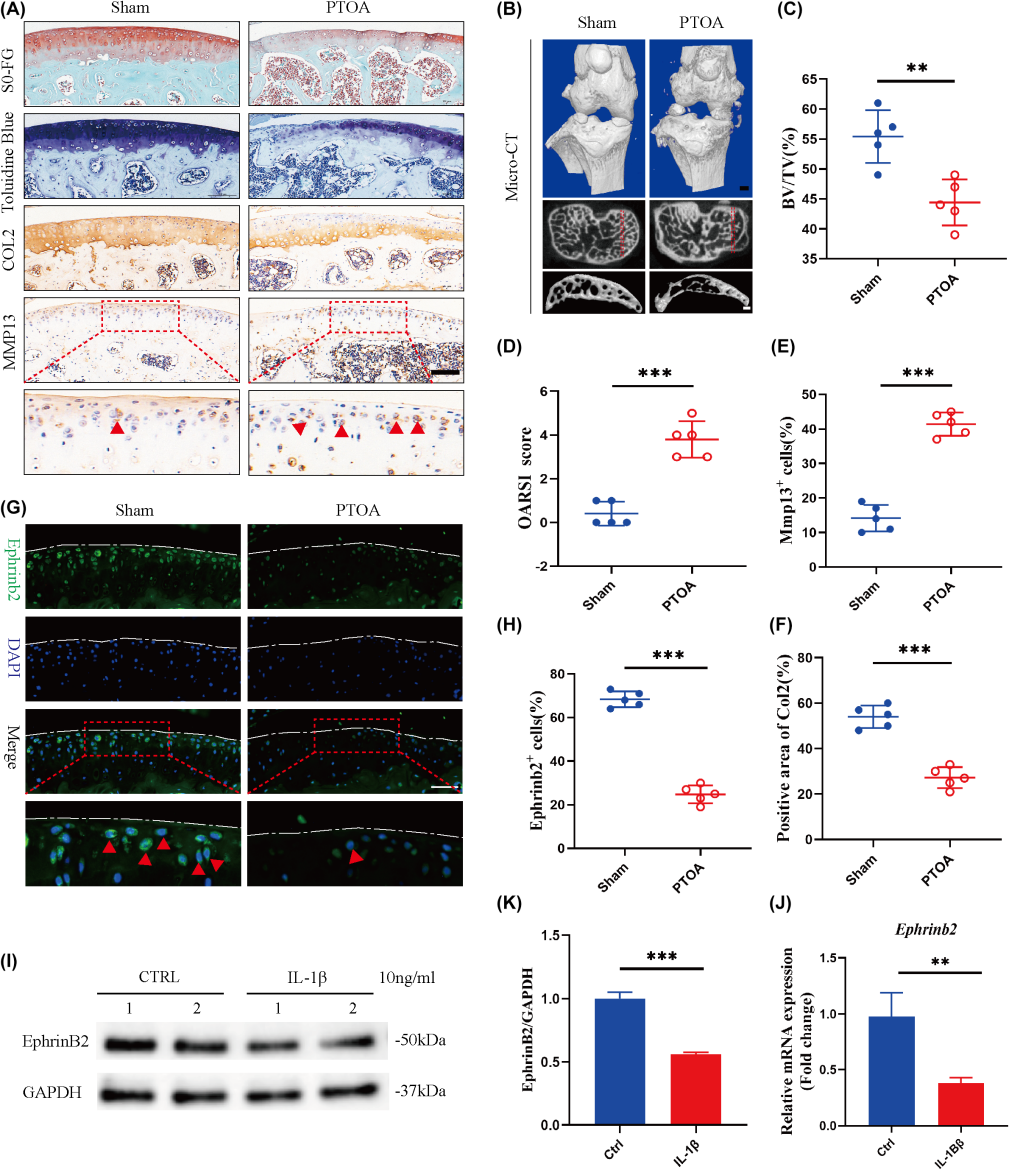
Expression of EphrinB2 in cartilage tissues of mouse knee joints. (A) Representative SO‐FG and toluidine blue staining and COL2 and Mmp13 immunohistochemistry staining of cartilage in Sham and PTOA mice. Scale bar:100 μm. (B) Micro‐CT 3D joint reconstruction of PTOA‐operated mice. The red dashed box in the transverse image indicates the ROI of the 3D reconstruction. Scale bar:100 μm. (C) Comparison of BV/TV in knee subchondral bone between Sham and ACLT mice 4 weeks after surgery. (D) OARSI score for assessing the degree of cartilage degeneration in the knee joints of Sham and ACLT mice. (E) Quantitative analysis of the ratio of MMP13‐positive chondrocytes in Sham and PTOA mice. (F) Quantification of the positive area of Col2 in cartilage of Sham and PTOA mice. (G, H) Representative immunofluorescence staining and EphrinB2‐positive ratio of chondrocytes in Sham and PTOA mice. Red arrows represent positive cells scale bar:100 μm. (I–K) The Western blot analysis of EphrinB2 protein expression in chondrocytes with IL‐1β treatment. (J) EphrinB2 mRNA expression in chondrocytes of mice treated with IL‐1β. All data were expressed as mean SD, (*n* = 5), *p < 0.05, **p < 0.01, ***p < 0.001.

We performed a surgical procedure to deliberately cause damage to the anterior cruciate ligament (ACL) in male C57BL/6 mice that were 3 months old. This was done in order to replicate a serious joint injury. Four weeks following ACLT, the mice showed notable alterations in the microstructure of the subchondral bone and deterioration of the cartilage matrix. Micro‐CT results showed that the parameter of BV/TV was significantly decreased in PTOA group compared to the sham group (Figure 2B,C). Besides, Safranin O‐Fast Green and toluidine blue staining demonstrated that ACLT induced severe PTOA in mice. After 4 weeks of ACLT, there was a notable loss of the cartilage matrix. The PTOA group exhibited a decrease in the area of Col2‐positive cells ranging from 15% to 39% as compared to the sham‐operated group. Similarly, the PTOA group exhibited a significant 18%–34% rise in the quantity of MMP13‐positive cells, accompanied by an increase in the OARSI score for cartilage damage (Figure 2A,E,F). Importantly, ACLT surgery impaired the EphrinB2 expression in cartilage (Figure 2G,H).

In addition, primary chondrocytes were isolated from the cartilage of femoral head of C57BL/6 mice (aged 2 weeks). The chondrocytes were cultured with a concentration of 10 ng/mL IL‐1β for 24 h. The RT‐qPCR and Western Blot results showed a decreased EphrinB2 expression in IL‐1β‐treated chondrocytes as demonstrated in Figure 2I–K.

### 3.3 Accelerated chondrocyte matrix degeneration in PTOA was observed following in vivo inhibition of Ephrinb2

**FIGURE 3:**
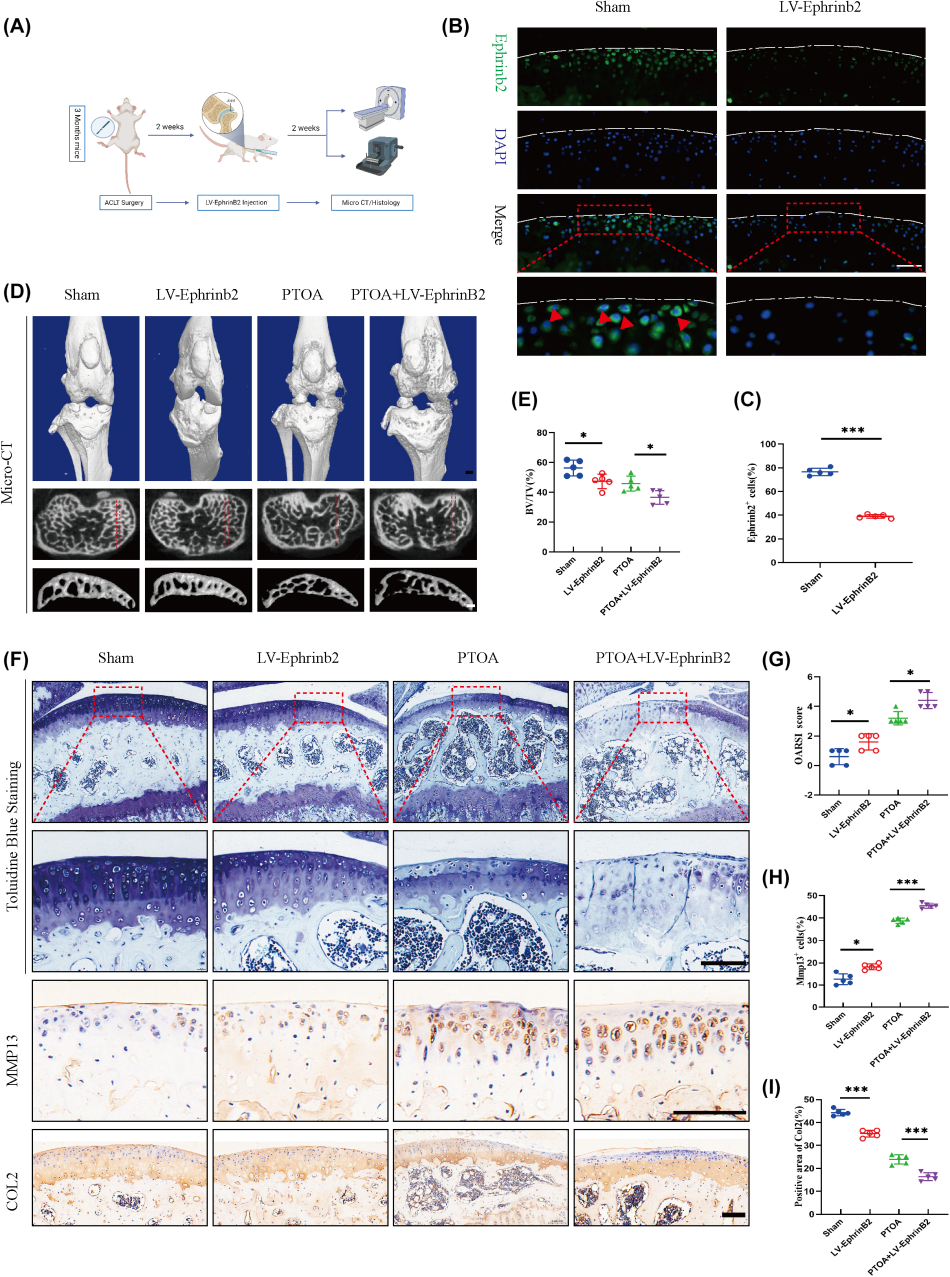
Inhibition of EphrinB2 in vivo accelerates matrix degeneration of chondrocytes in joints induced by trauma. (A) ACLT surgery and LV‐EphrinB2 intra‐articular infection in male C57BL/6J mice at 3 months of age. (B, C) EphrinB2 expression in articular cartilage of mice injected with LV‐EphrinB2 compared to the sham group. Red arrows indicate positive cells. Scale bar:100 μm. (D) Micro‐CT 3D joint reconstruction of mice in the Sham, LV‐EphrinB2, PTOA and PTOA+LV‐EphrinB2 groups at 4 weeks post‐operatively, and CT images of the subchondral bone in the transverse plane. The ROI of the 3D reconstruction is indicated by the red dashed box in the cross‐sectional image. Scale bar: 100 μm. (E) BV/TV in subchondral bone of the knee joint in Sham, LV‐EphrinB2, PTOA and PTOA+LV‐EphrinB2 mice at 4 weeks post‐operatively. (F, H, I) Representative toluidine blue staining and immunohistochemistry staining and quantification of COL2 and Mmp13 in knee joints of mice in the Sham, LV‐EphrinB2, PTOA and PTOA+LV‐EphrinB2 groups at 4 weeks postoperatively. (G) OARSI scores of cartilage degeneration in the knee joints of mice in the sham, LV‐EphrinB2, PTOA and PTOA+LV‐EphrinB2 groups at 4 weeks after surgery. All data were expressed as mean SD, (*n* = 5), *p < 0.05, **p < 0.01, ***p < 0.001.

To confirm the effect of EphrinB2 on chondrocyte matrix degeneration in mouse post‐traumatic knee joints, the anterior cruciate ligament was surgical cutting on 3‐month‐old mice, and then, 2 weeks later, an injection of LV‐EphrinB2 was performed to suppress the EphrinB2 expression in chondrocytes in vivo. All animals were sacrificed 4 weeks after the injury (Figure 3A). The immunofluorescence revealed that LV‐EphrinB2 effectively suppressed the EphrinB2 expression in chondrocytes in vivo, as demonstrated in Figure 3B,C. Micro‐CT analysis revealed that the BV/TV was significantly decreased in the PTOA group compared to sham group (Figure 3D,E). Toluidine blue staining indicated that the EphrinB2 suppression did not significantly reduce the cartilage matrix compared to the sham‐operated group. However, cartilage matrix degeneration was evident in the PTOA group, and EphrinB2 inhibition accelerated post‐traumatic articular chondrocyte matrix degeneration. Besides, the PTOA+LV‐EphrinB2 group exhibited obvious decrease of Col2 ranging from 2.67% to 11.75% and an significant increase in the number of MMP13+ positive cells ranging from 4% to 7.5% compared to the PTOA group (Figure 3F,H,I). Moreover, the suppression of EphrinB2 notably increased the OARSI score in the context of post‐traumatic cartilage injury (Figure 3G). These data proved that EphrinB2 inhibition could speed up the degradation of cartilage that have experienced post‐traumatic events.

### 3.4 Regulation of autophagy by EphrinB2 accelerates matrix degeneration and apoptosis in chondrocytes of PTOA

**FIGURE 4:**
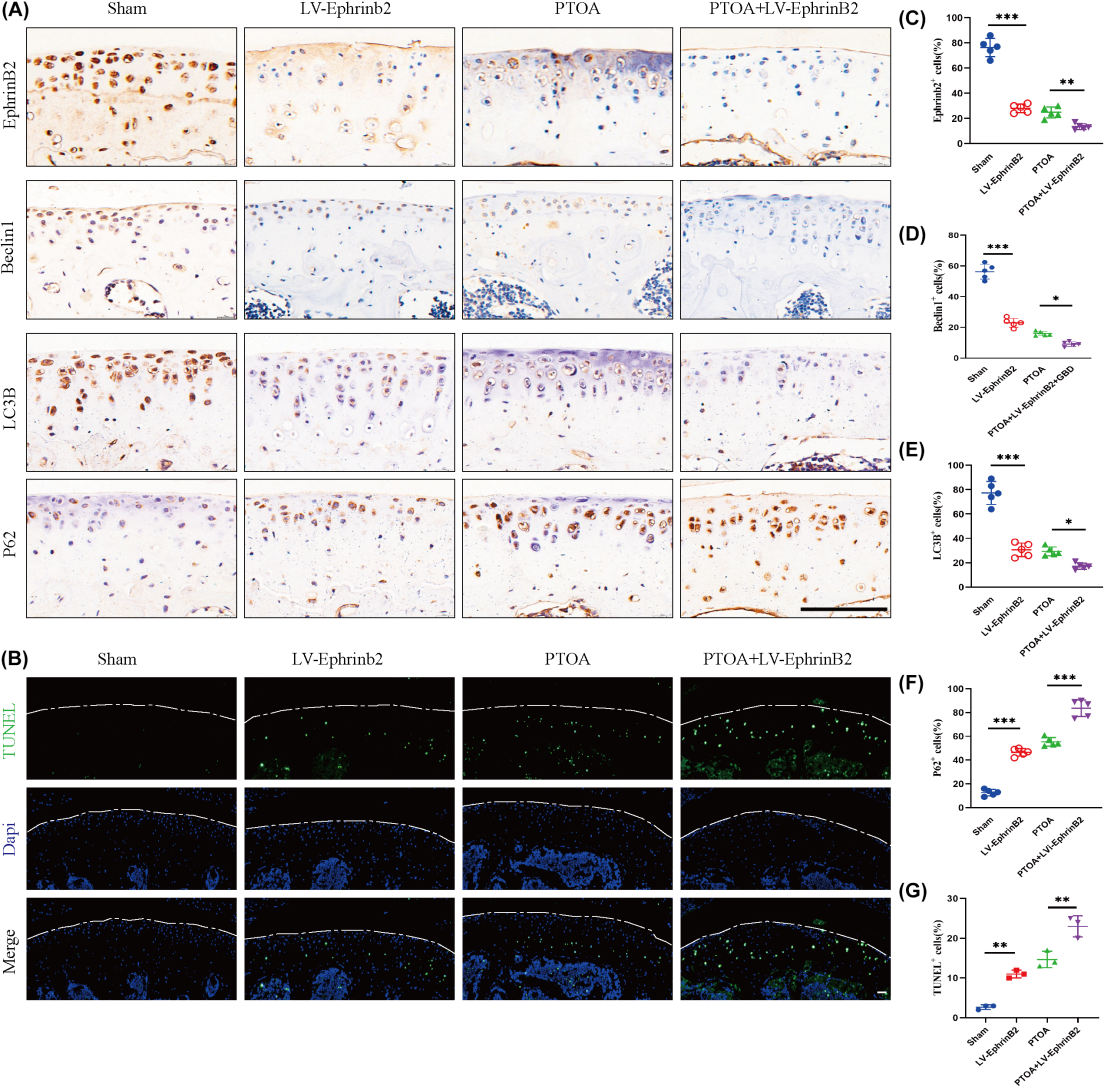
Inhibition of ephrinB2 in vivo accelerates chondrocyte apoptosis and matrix degeneration in an autophagy‐dependent manner. (A, C, D, E, F) Immunohistochemical staining and quantification of EphrinB2, Beclin1,LC3B, P62 in mouse cartilage. (B, G). TUNEL staining and quantification of mouse cartilage. All data were expressed as mean SD, (*n* = 5), *p < 0.05, **p < 0.01, ***p < 0.001.

Immunohistochemical staining demonstrated that autophagosome marker Beclin1 and LC3B was significantly decreased in PTOA group, as well as LV‐EphrinB2 group (Figure 4A,C–E). In the PTOA+LV‐EphrinB2 group, there was a considerable increase in the expression of the P62 protein, which is inversely related to autophagic activity (Figure 4A,F). In order to detect the impact of EphrinB2 suppression on the chondrocyte apoptosis in mice with PTOA, TUNEL staining was performed. The results showed a higher rate of chondrocyte apoptosis in the PTOA+LV‐EphrinB2 group compared to the PTOA animals (Figure 4B,G). These results suggest that EphrinB2 deficiency significantly inhibited chondrocyte autophagic flux and exacerbated post‐traumatic articular chondrocyte apoptosis.

### 3.5 In vitro inhibition of Ephrinb2 accelerates matrix degeneration in primary chondrocytes

**FIGURE 5:**
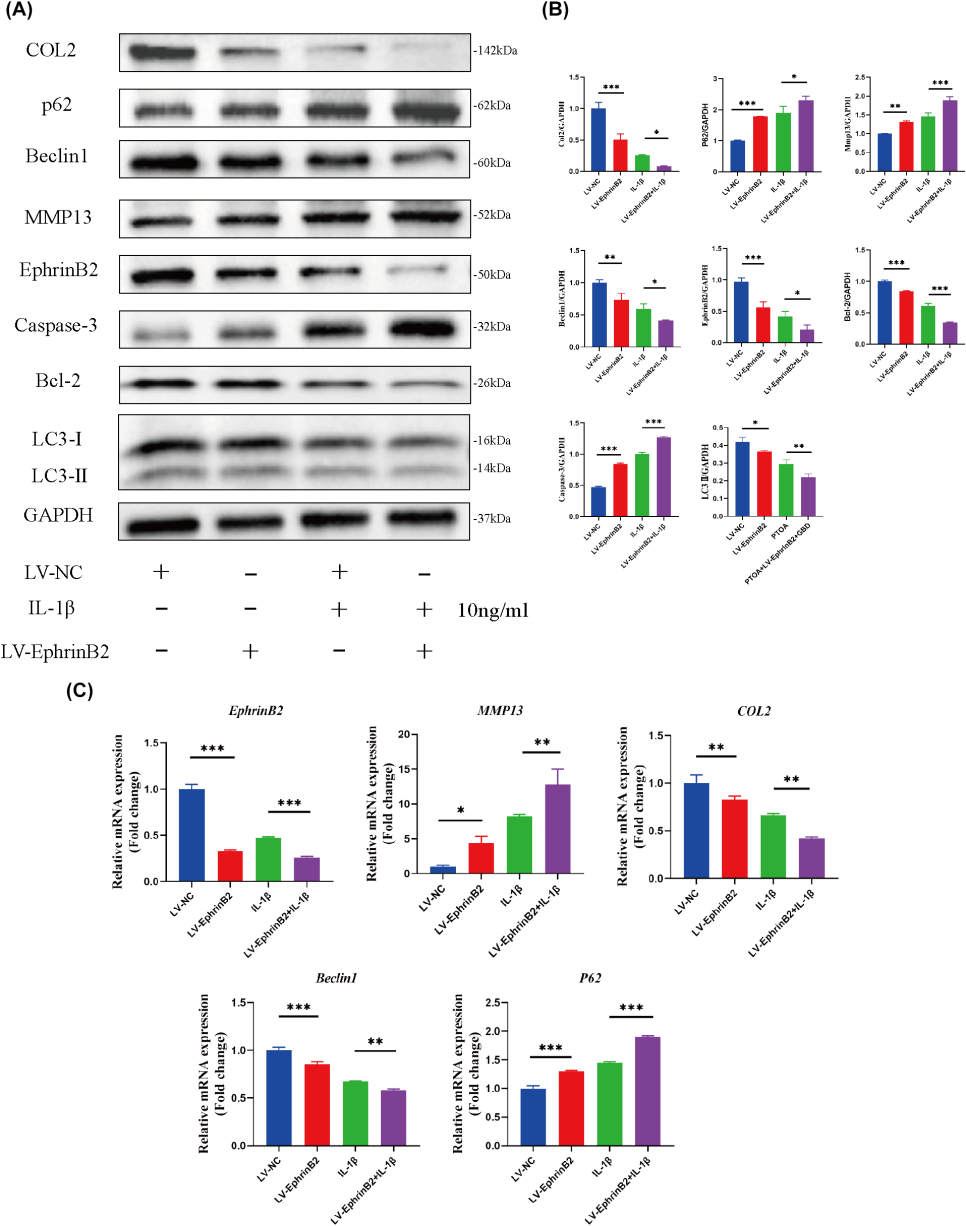
Ephrinb2 inhibition in primary chondrocytes accelerates autophagy‐dependent apoptosis and matrix degeneration. (A, B). Quantitative Western blot analysis of EphrinB2, Col2, Mmp13, LC3I/II, p62, Beclin1, Casepase‐3 and Bcl‐2 levels in primary chondrocytes after IL‐1β or LV‐EphrinB2 treatment. (C). EphrinB2, Col2, Mmp13, p62 and Beclin1 mRNA expressions in primary chondrocytes after IL‐1β or LV‐EphrinB2 treatment. All data were expressed as mean SD, (*n* = 3), *p < 0.05, **p < 0.01, ***p < 0.001.

To confirm the in vivo data, primary chondrocytes were transfected with lentiviral LV‐EphrinB2 to specifically knock down the Ephrin B2 expression (Figure 5C). RT‐qPCR and Western blotting analysis revealed that deficiency of EphrinB2 aggravated IL‐1β‐induced abnormal expressions of COL2 and MMP13 in chondrocytes. Furthermore, alterations in the autophagy level were proved in chondrocytes. The expression of Beclin1 and LC3I/II was reduced, as well as P62 was increased in chondrocytes that were stimulated by IL‐1β. This trend was even more obvious in the IL‐1β + LV‐EphrinB2 group. Besides, LV‐EphrinB2 treatment could further down‐regulate the anti‐apoptotic protein expression of Bcl‐2 based on IL‐1β induction. Additionally, the LV‐EphrinB2 + IL‐1β group showed further activation of caspase‐3, a key enzyme leading to apoptosis after stimulation by multiple inducers, compared to the IL‐1β‐induced group (Figure 5A). Therefore, these observations revealed that inhibiting EphrinB2 signalling in primary chondrocytes reduce chondrocyte autophagy levels and accelerate IL‐1β‐induced degeneration of the cartilage matrix and apoptosis.

## 4 DISCUSSION

This study initially identified a significant decrease in the expression of EphrinB2 in the articular cartilage of human patients with PTOA. To further investigate, we induced a PTOA phenotype in mice using ACLT surgery and then detected the EphrinB2 expression. This particular model was chosen because ACL injury is well acknowledged as the main risk factor for the development of PTOA.^22^, ^23^ The expression of EphrinB2 was markedly reduced in the knee cartilage of mice treated with ACLT. In order to inhibit the expression of EphrinB2 in chondrocytes of mice with PTOA, LV‐EphrinB2 was injected into the articular cavity. The results suggested that suppressing EphrinB2 in chondrocytes of mice with PTOA could hasten the deterioration of the chondrocyte matrix. We investigated changes in the levels of chondrocyte autophagy in PTOA mice and discovered that EphrinB2 deficiency resulted in a more profound reduction of autophagy in PTOA chondrocytes, which was accompanied by an increase in chondrocyte apoptosis.

The investigation of the Eph receptor and Ephrin ligand system in chondrocytes, together with their corresponding physiological and pathological mechanisms, is now in progress. EphB4 serves as the principal receptor for EphrinB2 and plays an important role in the complex process of bone remodelling and tissue transformation.^24^ In addition to EphrinB2, previous studies have revealed its involvement in chondrogenesis.^25^ Gladys Valverde‐Franco et al. have reported an osteoarthritis‐like phenotype in in mice that have been genetically modified to lack EphrinB2 specifically in their cartilage (EFNB2Col2KO). This phenotype was observed during early developmental abnormalities as well as the aging process of mice. The primary features of this phenotype were upregulated expression of collagen type X (COL10A1) in the growth plate region, disruption of cartilage hypertrophic regions and degradation of cartilage.^16^ Our study firstly reported that EphrinB2 deletion in mouse PTOA chondrocytes leads to increased OA characteristics.

Furthermore, EphrinB2 deficiency in PTOA chondrocytes accelerates cartilage matrix degeneration and promotes chondrocyte apoptosis by inhibiting chondrocyte autophagy. The EphrinB2 gene is associated with cellular autophagy.^26^, ^27^ Previous studies have suggested a correlation between EphrinB2, cellular autophagy, and protein kinase C. Sulfaphenazole treatment with cardiac cells could activate cellular autophagy and induce cardioprotective effects by involving EphrinB2 and protein kinase C.^28^ Furthermore, the deletion of the Eph ligand EphinB2 consistently induced autophagy‐mediated cell apoptosis in colon cancer cells.^29^ EphrinB2‐deficient osteocytes displayed more autophagosomes in vivo and in vitro, and EphrinB2‐Fc treatment suppresses autophagy in a RhoA‐ROCK dependent manner.^21^ Cellular autophagy is a complex process that interacts with multiple signalling pathways and molecular factors.^30^, ^31^, ^32^ The precise role of EphrinB2 in cellular autophagy is still unclear.

Autophagy and apoptosis are considered to have important functions in the normal and abnormal processes of chondrocytes in cartilage tissue.^33^ Suppression of autophagy can lead to an increase in the death of chondrocytes. This is caused by the accumulation of aberrant proteins and organelles, which in turn causes stress inside the cells and activates signals that lead to cell death.^34^ It is crucial to acknowledge that the interplay between autophagy and apoptosis can yield diverse outcomes in different cell types and pathological states. Thus, the impact of suppressing autophagy on chondrocyte apoptosis may be intricate and varied. Additional research is required to elucidate the precise mechanisms and impacts. Interestingly, we also observed an upregulation of TRAP+ cells in the subchondral bone of the tibial plateau of PTOA mice, and previous studies have also shown that ACLT can stimulate osteoclasts to become abnormally active.^35^ This suggests that abnormal bone metabolism in the subchondral bone is also an important reason for the rapid progression of PTOA.While the transfected and knockdown efficiency of LV‐EphrinB2 were confirmed in vivo, it should be noted that lentiviruses have limited specificity in targeting in living organisms due to their ability to infect a wide range of cells. This lack of specificity may lead to non‐specific effects.^36^ By improving the targeting accuracy and extending the duration of high knockdown specificity, it is possible to combine analytical approaches that have both high throughput and high sensitivity. This would enable the detection of alterations in EphrinB2 signalling and its mechanism involved in PTOA.

## 5 CONCLUSION

This is the first study showing that the expression of EprinB2 is reduced in chondrocytes during PTOA development. Additionally, blocking EprinB2 speeds up the course of PTOA by preventing chondrocyte autophagy. These findings provide a novel insight into the role of EprinB2 signalling in bone biology and propose EprinB2 as a potential cutting‐edge therapeutic target for the treatment of PTOA.

## AUTHOR CONTRIBUTIONS

**Zhengsheng Bao:** Conceptualization (lead); writing – review and editing (equal). **Pinger Wang:** Funding acquisition (equal); methodology (equal). **Yanan Li:** Methodology (supporting). **Jingyuan Wen:** Methodology (equal); supervision (equal); validation (equal). **Kaiao Zou:** Methodology (equal); software (equal). **Xu Wang:** Data curation (equal); software (equal). **Yang Yu:** Methodology (equal); supervision (equal). **Xuefeng Li:** Methodology (equal); resources (equal). **Yingquan Liu:** Data curation (equal); software (equal). **Hongting Jin:** Project administration (equal); supervision (equal); writing – review and editing (equal). **Lianguo Wu:** Conceptualization (equal); project administration (equal); supervision (equal). **Jun Ying:** Funding acquisition (equal); project administration (equal); supervision (equal); writing – review and editing (equal).

## FUNDING INFORMATION

This research was partially supported by the Natural Science Foundation of China (grant nos. 82104889, 82274280 and 82274550), Zhejiang Grants funded by the Provincial Natural Science Foundation of China (grant nos. LY22H270005 and LR23H0001), the Research and Development Program for “Pioneer” and “Leading Goose” of Zhejiang Province (grant no. 2024C03213). Science and Technology Program of Traditional Chinese Medicine in Zhejiang Province (grant nos. 2024ZR011 and 2024ZL514) Zhejiang University of Traditional Chinese Medicine Research Program Funding (grant no. 2023JKZKTS41).

## CONFLICT OF INTEREST STATEMENT

The authors declare no conflict of interest relevant to this article.

## Data Availability

The original contributions presented in the study are included in the article/supplementary materials, and further inquiries can be directed to the corresponding authors.
